# Alisol A Exerts Neuroprotective Effects Against HFD-Induced Pathological Brain Aging via the SIRT3-NF-κB/MAPK Pathway

**DOI:** 10.1007/s12035-023-03592-5

**Published:** 2023-09-02

**Authors:** Taotao Lu, Linlin Ding, Xiaoqing Zheng, Yongxu Li, Wei Wei, Weilin Liu, Jing Tao, Xiehua Xue

**Affiliations:** 1 College of Rehabilitation Medicine, Fujian University of Traditional Chinese Medicine, Fuzhou, 350112 China; 2 Fujian Key Laboratory of Rehabilitation Techniques, Cognitive Rehabilitation, Fuzhou, 350112 China; 3 The Affiliated Rehabilitation Hospital, Fujian University of Traditional Chinese Medicine, No 13, Hudongzhi Road, Fuzhou City, 350003 Fujian Province China

**Keywords:** High-fat diet, Aging, Microglial activation, Sirtuin-3, Nuclear factor-kappa B (NF-κB)

## Abstract

Chronic consumption of a high-fat diet (HFD) has profound effects on brain aging, which is mainly characterized by cognitive decline, inflammatory responses, and neurovascular damage. Alisol A (AA) is a triterpenoid with therapeutic potential for metabolic diseases, but whether it has a neuroprotective effect against brain aging caused by a HFD has not been investigated. Six-month-old male C57BL6/J mice were exposed to a HFD with or without AA treatment for 12 weeks. Behavioral tasks were used to assess the cognitive abilities of the mice. Neuroinflammation and changes in neurovascular structure in the brains were examined. We further assessed the mechanism by which AA exerts neuroprotective effects against HFD-induced pathological brain aging in vitro and in vivo. Behavioral tests showed that cognitive function was improved in AA-treated animals. AA treatment reduced microglia activation and inflammatory cytokine release induced by a HFD. Furthermore, AA treatment increased the number of hippocampal neurons, the density of dendritic spines, and the expression of tight junction proteins. We also demonstrated that AA attenuated microglial activation by targeting the SIRT3-NF-κB/MAPK pathway and ameliorated microglial activation-induced tight junction degeneration in endothelial cells and apoptosis in hippocampal neurons. The results of this study show that AA may be a promising agent for the treatment of HFD-induced brain aging.

**Supplementary Information:**

The online version contains supplementary material available at 10.1007/s12035-023-03592-5.

## Introduction

Excess consumption of a HFD and a sedentary lifestyle have undoubtedly exacerbated the obesity epidemic and the development of obesity-related metabolic disorders, which have far-reaching effects on human health [1]. As the population ages, scientists and clinicians have noted a significant association between a HFD and accelerated brain aging, including changes in brain structure, physiology, and function that are thought to lead to cognitive decline [2, 3]. In view of the expanding global epidemic of excessive HFD intake and the dementia crisis due to the rapid aging of the population, understanding the impacts of a HFD on brain aging, gaining further insights into possible underlying mechanisms, and developing effective therapeutic strategies are critical.

Although the pathogenesis of HFD-mediated accelerations in pathological brain aging is not yet clear, accumulating evidence has shown that neuroinflammation is a potential trigger of functional changes in the brain [4, 5]. A HFD has been proven to be a source of proinflammatory mediators. Increased intake of fatty acids (FAs) activates immune cells and inflammation in many organs, including adipose tissue, the liver, pancreas, and muscle [6]. In human and rodent models, a HFD typically increases the production of cytokines (interleukin (IL)-1β, IL-6, and tumor necrosis factor (TNF)-α), chemokines (chemokine ligand 2 (CCL2), motif chemokine 10 (CXCL10)), and the bacterial endotoxin lipopolysaccharide (LPS) [7]. These substances reach the cerebral microcirculation and pass through the damaged blood‒brain barrier (BBB) to induce neuroinflammation by activating microglia [8]. As resident macrophages, microglia perform primary immune surveillance and macrophage-like activities in the central nervous system (CNS), including the production of cytokines and chemokines. These cells control neuroinflammation and are sensitive to minute pathological changes in the CNS. Free fatty acids, cytokines, and chemokines bind to corresponding receptors on the microglial membrane to activate the immune response and further upregulate the expression of proinflammatory mediators, leaving the brain in a chronically inflamed state [9]. Sustained neuroinflammation disrupts neural homeostasis, which is linked to learning deficits, depression, and Alzheimer’s disease, accelerating pathological brain aging [10]. Evidence has shown that decreasing neuroinflammation by inhibiting microglial activation can ameliorate HFD-induced brain dysfunction.

Alisol A (AA) is an active triterpenoid component of *Alisma orientale (Sam.) Juz*. Recent studies have demonstrated that AA has therapeutic potential for the treatment of metabolic syndrome induced by a HFD [11]. Wang K et al. found that AA exerted hypolipemic, anti-inflammatory, and antiatherosclerotic effects on ApoE^−/−^ mice fed a HFD [12]. Another study showed that AA could markedly decrease lipid levels and alleviate glucose metabolism disorders and insulin resistance in mice with HFD-induced obesity [13]. These results suggest that AA may be a promising drug for the treatment of obesity and obesity-related metabolic disorders. However, there has been no detailed investigation of whether AA can reduce neuroinflammation and improve functional and structural brain impairment induced by a HFD.

Therefore, we first investigated the effects of AA on the neuroinflammatory response, microglial activation, and cognitive function in mice fed a HFD. We also investigated the cellular and molecular pathways underlying these effects by treating BV2 cells with the saturated fatty acid PA. Our results demonstrated that AA treatment alleviated pathological brain aging in mice compared to HFD mice. Moreover, our results revealed that inhibiting microglial activation may be one of the mechanisms by which AA mediates functional benefits in HFD mice.

## Materials and Methods

### Animals

All experimental procedures used in this study were approved by the Experimental Animal Ethics Committee of Fujian University of Traditional Chinese Medicine (SYXK (Min) 2019–0007)) and were conducted in accordance with the National Institutes of Health Guide for the Care and Use of Laboratory Animals. Six-month-old male C57BL6/J mice (*n* = 36) were purchased from Jiangsu Jicui Yaokang Biotechnology Ltd. (license SCXK2018-0008). The mice were housed five per cage and maintained on a 12-h light/dark cycle in a temperature-controlled environment with ad libitum access to food and water. After 1 week of acclimatization to the facility, the animals were randomly assigned to receive either normal chow (control group, n = 12), a high-fat diet (HFD group,* n* = 12, 60% fat Kcal%, MD12033, Medicience, Jiangsu, China), or a high-fat diet plus AA treatment (HFD + AA group, *n* = 12) for 12 weeks. AA (purity ≥ 98%, Yuanye Bio-Technology LTD, Shanghai, China) was diluted in normal saline and administered by gavage to the mice at a dose of 50 mg/kg/day for 12 weeks. We carefully chose a dose of AA based on a comprehensive analysis of previous studies [12, 13]. These studies indicating that 50 mg/kg falls within a therapeutically effective range. The behavioral tests and MRS commenced in the 10th week of the treatment period and were completed in the 12th week of the treatment period. After that, the mice were euthanized, and histological and biochemical tests were performed.

### Morris Water Maze Test (MWM)

The water maze system is composed of a water maze device, an automatic image acquisition system, and a software analysis system. The experiment is divided into two processes: acquisition training and probe training. In the acquisition phase, the pool was divided into four quadrants, and the platform was placed in the center of one of the quadrants. Each animal was given four trials per day for a total of 4 days and was allowed to swim to an escape platform. The mouse was placed facing the water with its head at the wall of the pool, and the time and distance taken by the animal to find the underwater platform were recorded. In the first few training sessions, if animal cannot find platform within 90 s, the animal was guided to the platform and allowed to stay on the platform for 10 s. The platform was removed in the probe training. The mice were placed in the pool on the opposite side of the original platform quadrant. The frequencies of the mice passing across the platform (removed) and the time of the mice swam in the platform (removed) area within 90 s were recorded as the detection index of spatial memory. Behavioral performance was tracked and analyzed with SuperMaze soft software (Xinruan Information Technology Co., Ltd., China).

### Novel Object Recognition Test (NORT)

The NORT includes four successive trials (trials 1–4, T1–T4). During T1, the mice were allowed to explore an empty arena for 5 min. The next day, the mice were placed into an open field containing two identical objects (A, B) and allowed to freely explore for 5 min (T2). After 1 h, T3 involved placing the mice in the open field arena containing A (familiar object) and C (novel object) and allowed to freely explore for 5 min. Twenty-four hours after T2, the mice were exposed to the familiar arena with two objects (familiar object A and novel object D) to test long-term recognition memory (T4). The time and frequencies that each mouse spent exploring the objects C and D (TC, TD) compared to the object A (TA) were recorded and traced using software (SuperMaze soft software, Xinruan Information Technology Co., Ltd., China).

### Magnetic Resonance Spectroscopy (MRS)

MRS has emerged as a possible sensitive measure of the structural and functional abnormalities associated with central nervous system dysfunction. To evaluate the changes reflected in mouse brain metabolism due to neuronal cell integrity and neuroinflammation, ^1^H-MRS scan was performed with a 9.4-T small animal MRI scanner (Biospec 94/30, Bruker, Germany) with a horizontal bore of 26 cm and a 12-cm internal diameter gradient coil insert. After anesthetization with 1.5% isoflurane, the mice were fixed with a custom-made holder including a tooth bar and ear bar to minimize head movement, and then the surface coil was fixed on the top of the mouse’s head. Then, the mice were sent to the receiving coil for scanning. During scanning, the mice were placed on a constant temperature blanket to keep them warm, and respiration and heart rates were measured simultaneously. Regions of interest (ROIs) were in the left hippocampus (1.0 mm × 1.0 mm × 1.0 mm), cortex (1.0 mm × 1.0 mm × 1.0 mm), and hypothalamus (2.0 mm × 1.0 mm × 1.0 mm) according to anatomical landmarks in T2-weighted fast-spin-echo images. A point-resolved selective sequence was used for signal acquisition with the following parameters: TR = 1500 ms, TE = 20 ms, and time = 6 min 24 s. The metabolites observed by MRS included creatine (Cr), N-acetylaspartate (NAA), myo-inositol (mI), choline (Cho), and macromolecules and lipids at 0.9 ppm (ML0.9). NAA is one of the more important compounds assessed on MRS, which is found in high concentrations in neurons and is a marker of neuronal health and density [14]. Changes in mI [15], Cho [16], and ML0.9 [17] have been reported in response to neuroinflammation or in inflammatory diseases.

In the analysis of MRS data, we used the software package TOPSPIN (version 3.1, produced by Bruker Biospin in Germany) to calculate the concentrations of various metabolites. In order to account for individual variations among the mice studied, we used the creatine (Cr) peak as our internal reference for the spectral data. Given that the levels of Cr remain relatively stable over several months, it allowed for a reliable comparison. Thus, the relative levels of the aforementioned metabolites were calculated based on their peak area ratios in comparison to this internal Cr reference.

### Golgi Staining

To visualize the morphological details of neurons, especially dendrites and dendritic spines, Golgi staining was performed using the Rapid GolgiStain ™ Kit (PK401, FD NeuroTechnologies, Columbia, USA) according to the manufacturer’s instructions. Briefly, the mice were anesthetized with isoflurane, and then the brain was removed from the skull and immersed in impregnation solution (equal volumes of solutions A and B). The impregnation solution was replaced the next day, and the sample was stored at room temperature for 2 weeks in the dark. Then, the brain tissues were transferred to solution C and stored at room temperature in the dark for 1 week, and the solution was replaced after 24 h. Next, the brains were sliced into coronal Sects. (100 μm) by using a vibrating blade microtome (Leica VT1000 S, Leica, Nussloch, German). The sections were rinsed in double-distilled water two times for 4 min each and then placed in the impregnation staining solution (one part solution D, one part solution E, and two parts double-distilled water) for 10 min. The sections were dehydrated with sequential rinses of 50%, 75%, 95%, and 100% ethanol for 4 min each. Finally, the sections were cleared with xylene to make the tissues transparent. The sections were observed using an optical microscope (Model Eclipse Ci-L, 718345, Nikon, Japan) and analyzed using ImageJ software (ImageJ, version 6.0, NIH, Bethesda, MD).

### Immunohistochemistry (IHC)

After the behavioral tests, the anesthetized rodents were transcardially perfused with PBS and then 4% paraformaldehyde, and the brain was harvested. The brain was fixed in 4% paraformaldehyde and embedded in paraffin to prepare sections with a thickness of 4 μm. IHC was performed according to the instructions of the UltraSensitive SP (Mouse/Rabbit) IHC Kit (KIT-9720, MXB Biotechnologies, Fujian, China). After deparaffinization and rehydration, the slices were immersed in antigen retrieval solution, heated in a microwave oven for 15 min, and then washed with PBS three times after 2 h of natural cooling. The slices were incubated in 3% H_2_O_2_ for 15 min to eliminate the effect of endogenous hyperoxide and then washed three times with PBS solution. Next, the slides were blocked with blocking buffer at room temperature for 1 h and then incubated with primary antibodies (Iba1, 1:200, 10,904–1-AP, Proteintech) overnight at 4 °C. The peroxidase-labeled polymer secondary antibody was added dropwise and incubated for 1 h at 37 °C, after which the slides were washed with PBS and then incubated with DAB (MXB biotechnology, Fujian, China) for 1 min at room temperature until a brown color developed. Finally, cover glasses were mounted onto glass slides with neutral resin and visualized using an optical microscope.

### Cell Culture

The microglial cell line (BV2, BNCC337749, Beina Biology, Beijing, China) and hippocampal neuronal cell line (HT22, BNCC337749, Beina Biology, Beijing, China) were cultured in high glucose Dulbecco’s modified Eagle’s medium (DMEM/H, C11995500BT, Gibco) supplemented with 10% (v/v) fetal bovine serum (FBS, F8318, Sigma) and 1% penicillin/streptomycin and were passaged every 2 to 3 days. Mouse brain microvascular endothelial cells (bEnd.3, CC-Y2019, Shanghai Jining Industrial LTD, Shanghai, China) were cultured in DMEM/H with 15% (v/v) FBS and 1% penicillin/streptomycin; the culture medium was changed every 2 days, and the cells were passaged every 3 to 4 days. bEnd.3 cells below passage 9 were used in the experiments. The three types of cells were cultured at 37 °C in a humidified 5% CO_2_ atmosphere.

### Palmitate Intervention and AA Treatment

AA was dissolved in dimethyl sulfoxide (DMSO), prepared into a 200 μM stock solution with serum-free medium and diluted in complete medium for use. Palmitate (PA, P9767, Sigma, USA) is a representative saturated fatty acid. A 20 mM stock solution was made by dissolving PA (0.334 g) in 3 mL of sterile water at 70 °C, which was then mixed with 3 mL of prewarmed 40% bovine serum albumin. The solutions were filtered using a 0.22-mm syringe filter, stored in a refrigerator at 4 °C, and diluted with complete culture medium for use.

### Generation of Stable Transduction Cell Lines

To further verify the mechanism by which AA inhibits PA-induced BV2 cell activation, three nonoverlapping anti-SIRT3 short hairpin RNA (ShRNA) oligonucleotides and lentivirus-mediated SIRT3 overexpression vectors were designed by Zolgene Biotech (Fujian, China). BV2 cells were transfected with the SIRT3 lentivirus according to the manufacturer’s instructions. In brief, BV2 cells in the logarithmic growth phase were inoculated in 6-well plates at a density of 5 × 10^4^ cells/well. For stable infection, the cells were treated for 24 h with complete DMEM containing the lentivirus in the presence of 5 μg/mL polybrene when the cells reached 50% confluence. After 6 h, the cells were supplemented with 1 mL of DMEM and incubated overnight at 37 °C. Twenty-four hours after infection, the lentivirus-containing medium was changed to normal culture medium, and stably expressed cells were selected with puromycin (final concentration, 1 µg/mL). For all lentivirus experiments, the virus was used at a multiplicity of infection (MOI) of 30.

### Culturing HT22 and bEnd.3 Cells with Different Types of BV2-Conditioned Medium

After being cultured under normal culture conditions to 70–80% confluence, HT22 and bEnd.3 cells were cultured with different types of BV2-conditioned medium for another 24 h. The groupings in experiments involving BV2-conditioned medium were based on BV2 cell treatments. The sources of BV2-conditioned medium included medium from BV2 cells treated with normal DMEM (BV2-Medium), BV2 cells stimulated with PA for 8 h (BV2-PA), BV2 cells that were pretreated with AA for 24 h and then stimulated with PA for 8 h (BV2-PA + AA), shRNA-SIRT3 BV2 cells that were pretreated with AA for 24 h and then stimulated with PA for 8 h (BV2-PA + AA + Sh-SIRT3), and shRNA-NC BV2 cells that were pretreated with AA for 24 h and then stimulated with PA for 8 h (BV2-PA + AA + Sh-NC).

### Cell Viability Assay

Cell viability was assessed using Cell Counting Kit-8 (CCK-8; AR1160, BOSTER, Wuhan, China) according to the manufacturer’s instructions. Briefly, cells were seeded into 96-well plates in complete DMEM and incubated at 37 °C. At specific time points, CCK-8 reagent (10 μL/well) was added to each well and incubated for an additional 1 h at 37 °C in the dark. The absorbance was measured at 450 nm using a microplate reader (Bio-Tek, Winooski, VT, USA).

### RNA Purification and Quantitative Real-Time PCR

Total RNA was extracted from cells using TRIzol reagent (R401-01, Vazyme, Nanjing, China). The RNA was reverse transcribed into cDNA by using the PrimeScript RT reagent kit (RR047A, Takara, Shiga, Japan). Real-time PCR was performed using SYBR Premix (RR420A, Takara, Shiga, Japan) according to the manufacturer’s instructions in an ABI 7500 Fast Real-Time PCR System. Glyceraldehyde 3‐phosphate dehydrogenase (GAPDH) was used for normalization. Relative mRNA expression was determined using the cycle threshold (CT) formula 2^−△△CT^, where △CT = [CT (target gene) – CT (GAPDH)]. The specific primer sequences used in this study are detailed in the supplementary material (Table S1).

### Immunofluorescence Assay

The cells were blocked and incubated with primary antibodies against CD16/32 (1:300, Cell Signaling, Cat# 80366), ZO-1 (1:300, Proteintech, Cat# 21773–1-AP), Occludin (1:300, Proteintech, Cat# 66378–1-Ig), and claudin-5 (1:200, Abcam, Cat# 15106) overnight at 4 °C and then incubated with fluorescent-conjugated secondary antibodies for 90 min at 37 °C. Nuclei were stained with DAPI for 5 min. Immunofluorescence was visualized and acquired using a confocal microscope (LSM710, ZEISS, Germany).

### Western Blot (WB) Analysis

Relative protein levels in whole brain samples were determined by WB analysis. The protein samples were separated and transferred to a polyvinylidene difluoride (PVDF) membrane. The PVDF membrane was incubated with primary antibodies at 4 °C overnight. The primary antibodies and their concentrations were as follows: NF-κB (1:1000, Cell Signaling, Cat# 8242), p-NF-κB (1:1000, Cell Signaling, Cat# 3033), MAPK (1:1000, Cell Signaling, Cat# 9212), p-MAPK (1:1000, Cell Signaling, Cat# 4631), CSF1R (1:100, SANTA CRUZ, Cat# 365719), IL-1β (1:1000, Abcam, Cat# Ab9722), TNF-α (1:1000, Cell Signaling, Cat# 11948), iNOS (1:2000, Proteintech, 18985–1-AP), Bcl-2 (1:1000, Proteintech, Cat# 26593–1-AP), Bax (1:2000, Proteintech, Cat# 50599–2-lg), Cle-caspase-3 (1:1000, Proteintech, Cat# 19677–1-AP), GAPDH (1:7000, Proteintech, Cat# 60004–1-lg), claudin-5 (1:500, Abcam, Cat# 15106), ZO-1 (1:1000, Proteintech, Cat# 21773–1-AP), and occludin (1:800, Proteintech, Cat# 66378–1-Ig). After being incubated with horseradish peroxidase-conjugated secondary antibodies at room temperature for 2 h, the protein bands were visualized with an enhanced chemiluminescence kit (MA0186, Meilunbio). The protein bands were imaged with the Bio-Image Analysis system (Bio-Rad Laboratories, Inc.) and analyzed with ImageJ software.

### Enzyme-Linked Immunosorbent Assay (ELISA)

TNF-α and IL-1β concentrations in differentially treated BV2 cell culture supernatants were measured using ELISA kits (VAL609, VAL601, R&D SYSTEMS) according to the manufacturer’s protocol. The results were calculated by measuring the absorbance at a wavelength of 450 nm using a multiplate reader.

### Endothelial Monolayer Permeability Assay

bEnd.3 cells were grown to confluence on 24-well Transwell permeable support plates (3413, Corning, NY, USA, 0.4-µm pore size, polycarbonate membrane, 6.5-mm insert diameter). After the cells were cultured with different types of BV2-conditioned medium for 24 h, 20 µg/mL FITC-dextran (70 kDa) was added to the top chamber. After 1 h of equilibration, dextran transendothelial flux was measured using a fluorescence microplate reader at an excitation wavelength of 493 nm and an emission wavelength of 518 nm.

### Annexin V-FITC Apoptosis Assay

After HT22 cells were cultured with different types of BV2-conditioned medium for 24 h, apoptosis was examined by using an Annexin V-FITC Apoptosis Detection Kit (KGA105-KGA108, KeyGEN BIOTECH, Jiangsu, China) according to the manufacturer’s instructions. The percentage of apoptotic cells was analyzed by flow cytometry (Becton Dickinson).

### Statistical Analysis

All experiments were performed with at least three independent replicates. Analyses were performed using one-way analysis of variance (ANOVA) with the least significant difference test for normally distributed data. Additionally, two-way repeated measurement ANOVA was performed to compare repeated measurement data. We used IBM SPSS statistics software (Version 23.0. Armonk, NY, USA) for statistical analysis. All data are presented as the mean ± SEM. A *p* value < 0.05 was defined as statistically significant.

## Results

### AA Ameliorated Cognitive Deficits in Mice Fed a HFD

**Fig. 1:**
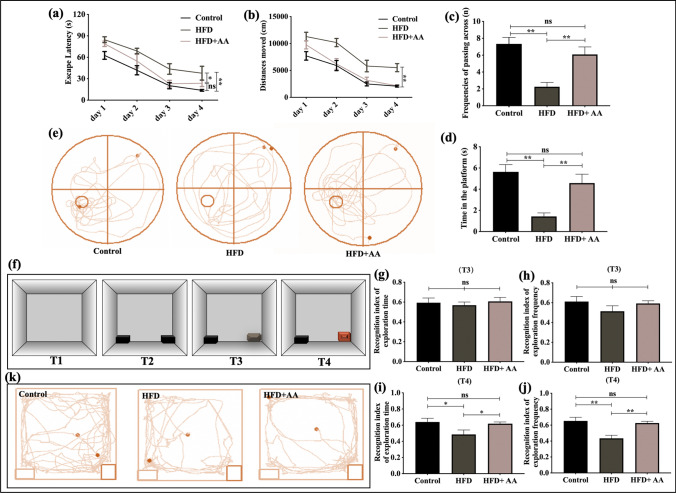
AA treatment improved cognitive impairment in mice with long-term HFD feeding. **a** The time the mice took to find the underwater platform (escape latency). **b** The distance of the mice traveled to find the underwater platform (distance moved). **c** The frequencies of the mice passing across the platform (removed) in probe trial of the MWM. **d** The time of the mice swam in the platform (removed) area in the probe trial of the MWM. **e** Representative swimming trajectory of the mice in the probe trial of the MWM. **f** Schematic diagram of the trials (T1–T4) and objects used in the novel object recognition task. **g** The time the mice spent exploring the novel object compared to the novel object plus the familiar object (recognition index of exploration time) during T3. **h** The frequencies of exploring the novel object compared to the novel object plus the familiar object (recognition index of exploration frequency) during T3. **i** The time the mice spent exploring the novel object compared to the novel object plus the familiar object (recognition index of exploration time) during T4. **j** The frequencies of exploring the novel object compared to the novel object plus the familiar object (recognition index of exploration frequency) during T4. **k** Representative exploration trajectory of the mice during T4. The data are presented as the means ± SEMs. *n* = 12 for each group. Statistical significance is indicated as *p < 0.05, **p < 0.01

Cognitive function was evaluated by the MWM and NORT. During the acquisition training phase of the MWM, we recorded the time (escape latency) and distance (distances moved); it took the mice to reach the escape platform. During the probe training phase of the MWM, we recorded the frequencies of the mice passing across the platform (removed) and the time of the mice swam in the platform (removed) area within 90 s. As shown in Fig. 1a and b, compared the control mice, HFD mice showed higher escape and total distances moved in the acquisition training, whereas escape and path length were decreased in the AA intervention group (*p* < 0.05). There was a notable difference in swimming traces among the three groups (Fig. 1e). The HFD group mainly swam on the edge of the pool and had a lower frequency of passing across the platform and reduced time in the platform area compared with the control group. The HFD + AA group showed an increase in platform crossings. Moreover, AA-treated mice showed longer swimming times in the position of the removed platform in contrast to HFD mice that received no treatment (*p* < 0.05, Fig. 1c, d). The NORT demonstrated that the recognition index of novel objects between the three groups during T3 was not significantly different (*p* > 0.05, Fig. 1g, h). However, the HFD group exhibited impaired recognition memory during T4, which was evident from their lack of preference to explore the novel object (Fig. 1i, j). In contrast to the HFD group, the control group and HFD + AA group showed proficiency for novel object recognition, as represented by an increased recognition index (*p* < 0.05). These results demonstrated that a chronic HFD is associated with cognitive impairment and that alisol A intervention ameliorated cognitive damage.

### Metabolic Changes in Mice Chronically Exposed to a HFD

**Fig. 2:**
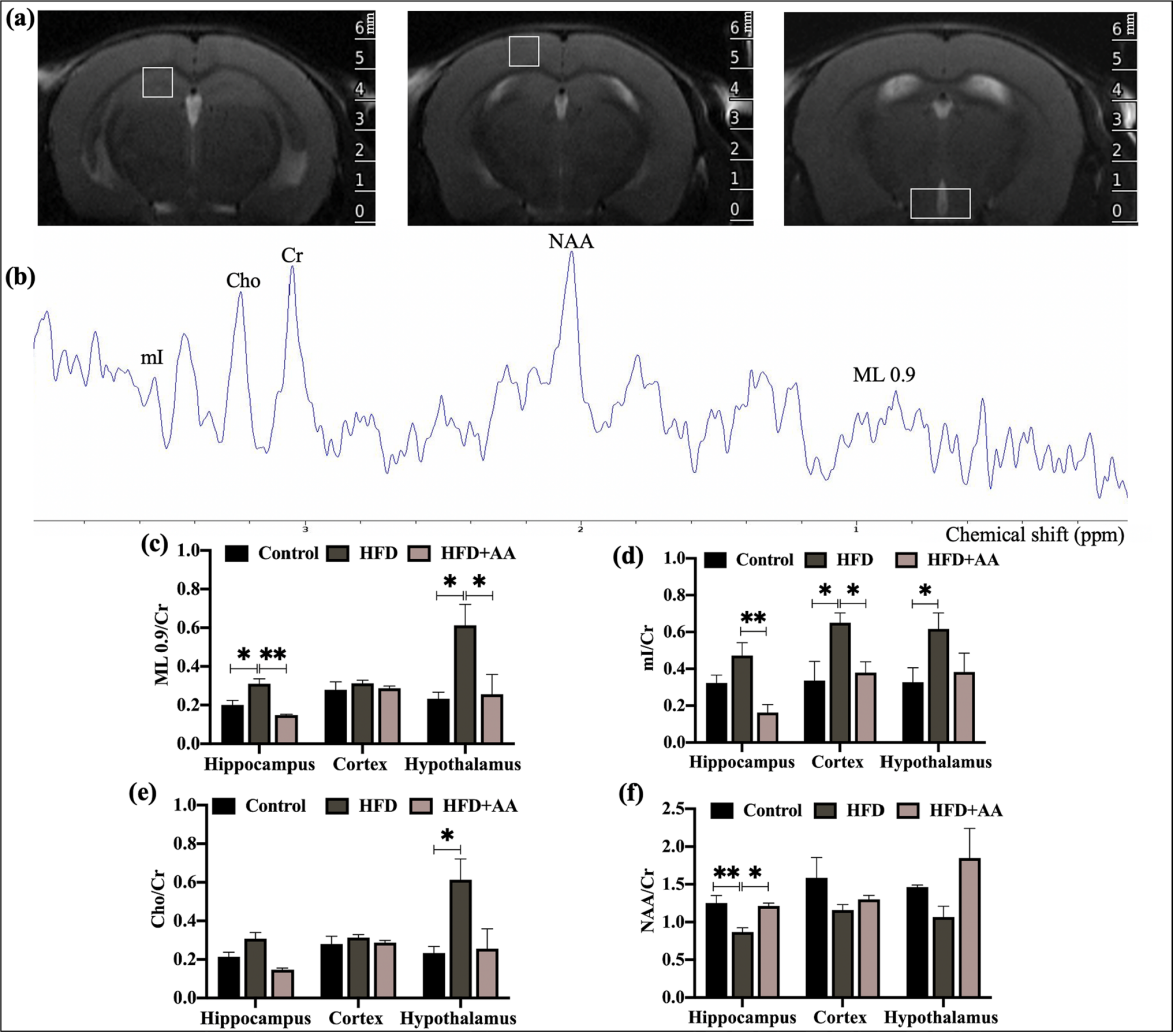
The neurochemicals and metabolic changes in the brains of mice. **a** The region of interest in the hippocampus, cortex, and hypothalamus. **b** Typical spectra of the hippocampus. **e**–**g** Changes in the levels of the metabolites NAA, mI, ML 0.9, and Cho (*n* = 3–6). Cr was used as an internal reference to measure the levels of the other metabolites. The data are presented as the means ± SEMs

In this study, MRS was used to quantify brain metabolite profiles in a noninvasive manner. The ROI and typical spectra are shown in Fig. 2a and b. Changes in mI [15], Cho [16], and ML0.9 [17] have been reported in response to neuroinflammation or in inflammatory diseases. As shown in Fig. 2c, the mice responded to HFD feeding with an increase in ML0.9 in the hypothalamus and hippocampus (*p* < 0.05), which was not observed in the control and HFD + AA groups, although there was no obvious change in ML0.9 in the cortex in the three groups (*p* > 0.05). In the hypothalamus, hippocampus, and cortex, the concentration of mI was increased dramatically in HFD mice compared with controls (Fig. 2d) (*p* < 0.05). AA treatment reduced the concentration of mI in this area. Additionally, the concentration of Cho was increased in the hypothalamus of HFD mice (*p* < 0.05, Fig. 2e). NAA concentrations are measured with MRS and often viewed as markers of neuronal integrity [18]. Compared to the control group, the HFD group exhibited a decrease in NAA in the hippocampus (*p* < 0.05, Fig. 2f) but not in the hypothalamus or cortex. The HFD + AA group exhibited increased NAA concentrations in the hippocampus. These results indicated that HFD exposure resulted in changes in brain metabolic profiles associated with inflammatory processes and the integrity of neurons, whereas AA treatment was conducive to maintaining metabolic homeostasis.

### AA Inhibited Neuroinflammation in Mice Fed a Long-Term HFD

**Fig. 3:**
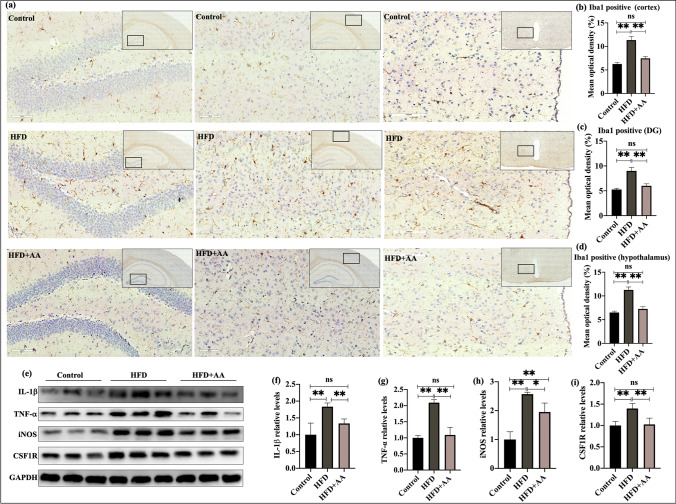
AA inhibited neuroinflammation in mice fed a HFD for 12 weeks. **a** Iba1 + immunostaining in the hippocampus, cortex, and hypothalamus. **b**–**d** Quantification of Iba1 + immunostaining in the hippocampus, cortex, and hypothalamus (*n* = 4). **e** Representative western blots of IL-1β, TNF-α, iNOS, and CSF1R. **e**–**h** Quantitative analysis of IL-1β, TNF-α, iNOS, and CSF1R levels in the brains of mice (*n* = 3). The data are presented as the means ± SEMs. Statistical significance is indicated as *p < 0.05, **p < 0.01

As resident macrophages, microglia are the main effectors of the inflammatory response of the CNS and play an active role in HFD-induced brain aging. Following 12 weeks of HFD feeding, inflammation developed in the CNS (Fig. 3). Iba1 was used to stain microglia. The results showed that 12 weeks of HFD feeding resulted in significant microglial activation in the cortex, dentate gyrus of the hippocampus, and hypothalamus; and Iba1 immunohistochemical staining was enhanced (*p* < 0.05, Fig. 3a–d). In addition, proinflammatory cytokines, including TNF-α, IL-1β, inducible nitric oxide synthase (iNOS), and colony-stimulating factor 1 receptor (CSF1R, which directly controls the development, survival, and maintenance of microglia and plays a pivotal role in neuroinflammation), were increased in the brains of HFD-fed mice (*p* < 0.05, Fig. 3e–i). Moreover, treatment of HFD-fed mice with AA suppressed the effects of a HFD (*p* < 0.05). In summary, AA can decrease neuroinflammation in mice fed a long-term HFD.

### AA Reduced HFD-Induced Damage to Neurons and the Blood‒Brain Barrier

**Fig. 4:**
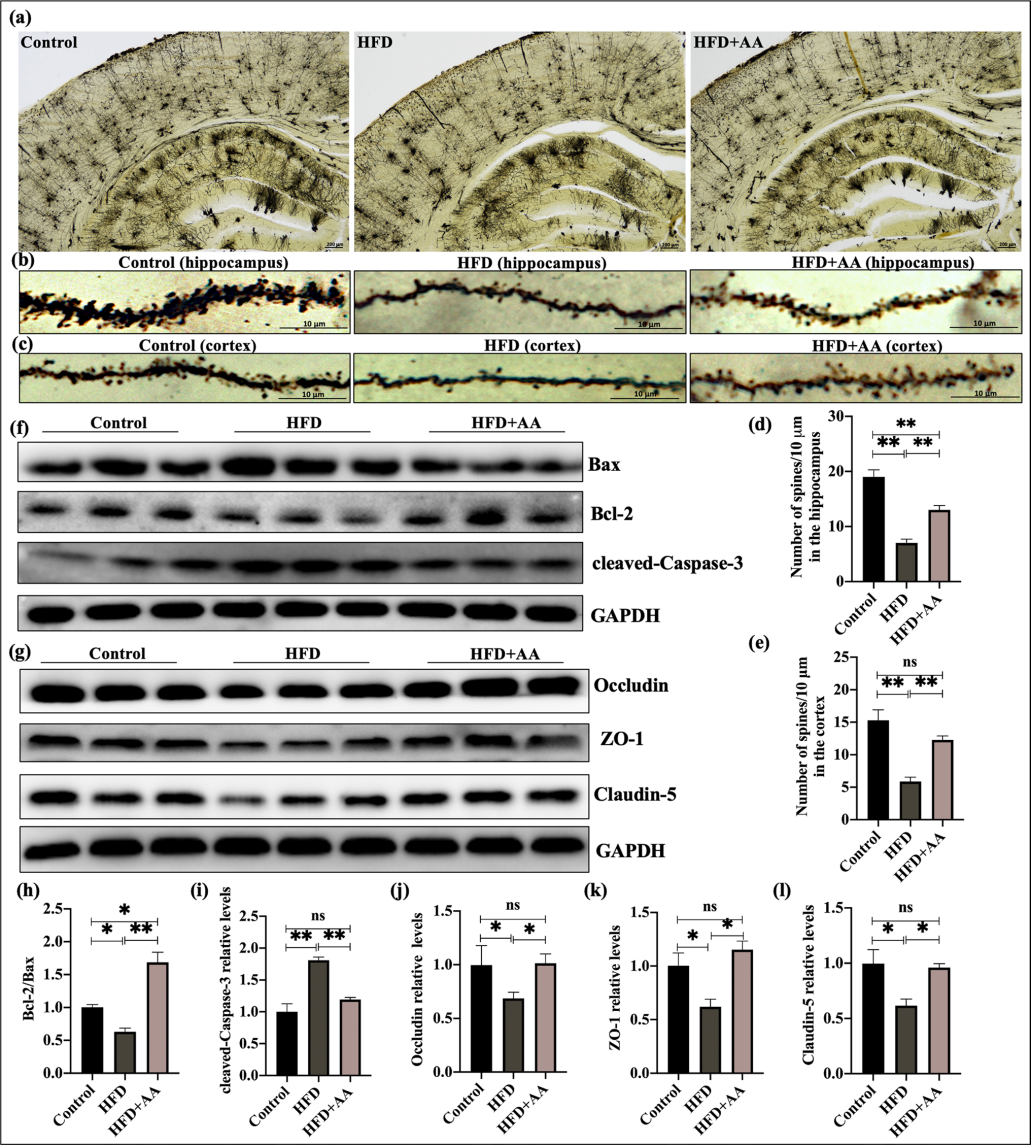
AA alleviated neurovascular injury in mice fed a HFD for 12 weeks. **a** Representative Golgi staining (4 × magnification) of the control, HFD, and HFD + AA groups. **b**, **c** Golgi staining (100 × magnification) was used to observe the density of dendritic spines in hippocampal and cortical neurons. **d**, **e** Quantification of the number of spines in the hippocampus and cortex (*n* = 4). **f**, **g** Representative western blots of apoptosis-related proteins (Bax, Bcl-2, and cle-caspase-3) and tight junction proteins (occludin, ZO-1, and claudin-5). **h**–**l** Quantitative analysis of Bcl-2/Bax, cle-caspase-3, occludin, ZO-1, and claudin-5 levels in the brains of mice (*n* = 3). The data are presented as the means ± SEMs. Statistical significance is indicated as *p < 0.05, **p < 0.01

Our results indicate that 12 weeks of HFD feeding induced neuroinflammation in the brains of mice. Research has acknowledged sustained neuroinflammation as a potential trigger of functional changes in the BBB and neurons [2]. Therefore, we further observed neuronal morphology by Golgi staining and the expression of apoptosis-related protein and tight junction (TJ) proteins by WB analysis in the brain of each group. As shown in Fig. 4a, hippocampal neurons were significantly decreased and arranged irregularly in the HFD group compared with the control group. The mice that received AA showed an increase in the number of hippocampal neurons that were arranged neatly and regularly. We also found that dendritic spines in the hippocampus and cortical region neurons were dense in the control group, while in the HFD group, the dendritic spines fell off and were atrophied (*p* < 0.05, Fig. 4b–e). However, the number of dendritic spines in the HFD + AA group was higher than that in the HFD group (*p* < 0.05). Figure 4 f, h, and i show that there was a marked increase in cleaved caspase-3 expression and the Bax/Bcl-2 ratio in the HFD group compared to the control group (*p* < 0.05), which was alleviated by AA treatment. In addition, there was a decrease in TJ (occludin, ZO-1, and claudin-5) expression in the brains in the HFD group compared with the control group and the HFD + AA group (*p* < 0.05, Fig. 4g, j–l). In summary, we observed that AA protected against HFD-induced neuronal and BBB damage.

### AA Attenuated Inflammatory Responses in PA-Induced BV2 Cells

It is thought that HFD-induced neuroinflammation plays a key role in brain function damage and that the inflammatory response is mediated by activated microglia. Therefore, properly inhibiting the activity of microglia is an effective method to prevent pathological brain aging induced by HFD. To verify the effect of AA on the activation of microglia induced by high fat in vitro, BV2 cells were exposed to the representative saturated fatty acid PA, which was used to induce microglial activation. As shown in Supplementary Figs. 1a and c, after BV2 cells were treated with PA for 8 h, optical microscopy and CCK-8 assays demonstrated that PA (175 mM) induced obvious M1-type polarization in BV2 cells without affecting cell viability; the cells were rounded or oval, while untreated BV2 cells were spindle-shaped or elongated. As shown in Supplementary Fig. 1b, 5 to 80-μM AA did not affect cell viability.

**Fig. 5:**
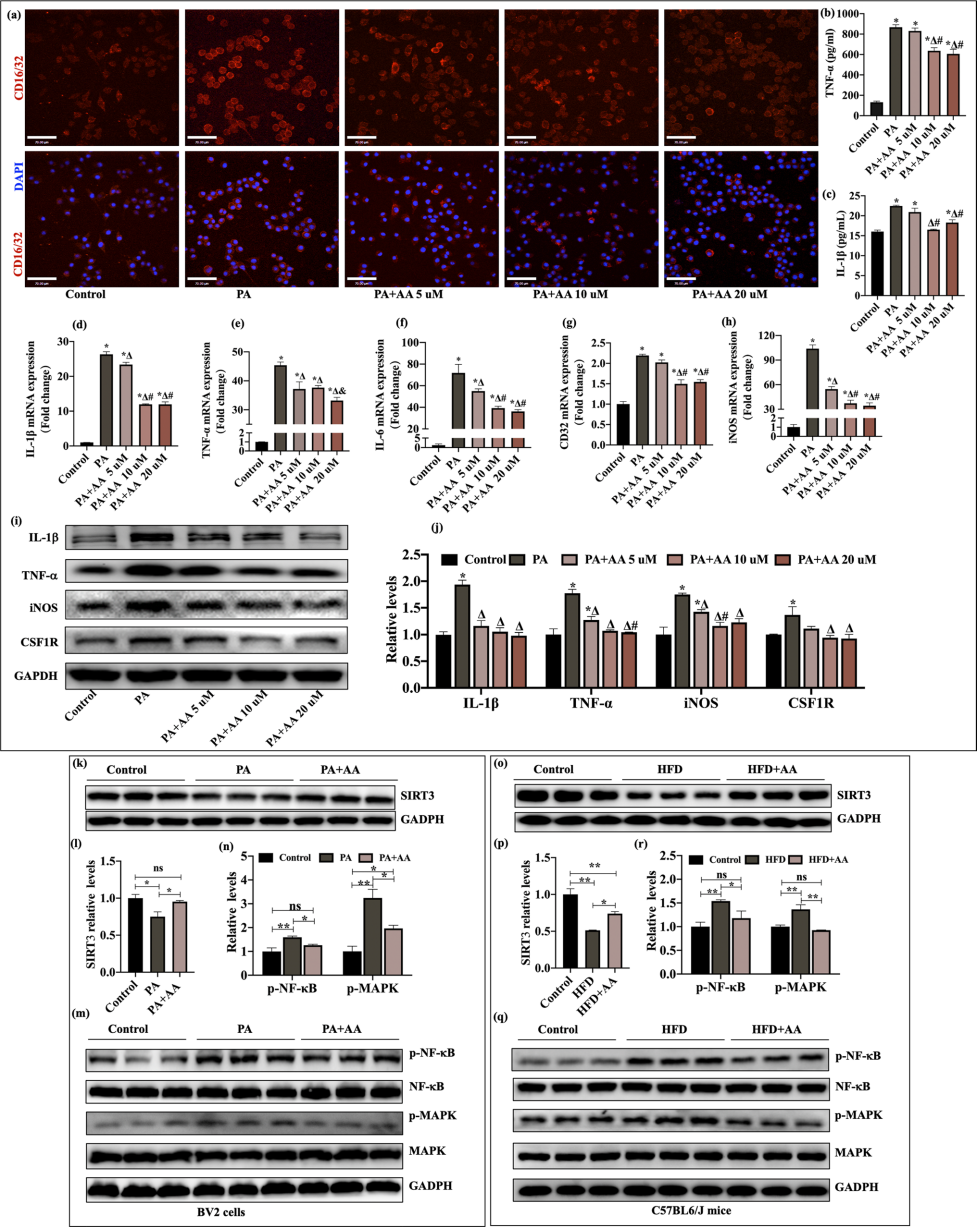
AA attenuated inflammatory responses in PA-induced BV2 cells. BV2 cells were pretreated with alisol A and then stimulated with PA. **a** Representative immunostaining of the M1 macrophage surface marker CD16/32 (red) in each group. **b**, **c** TNF-α and IL-1β levels in the culture supernatant were measured by ELISA (*n* = 3). **d**–**h** The mRNA expression of IL-1β, TNF-α, IL-6, CD32, and iNOS in each group (*n* = 3). **i** Representative western blots of IL-1β, TNF-α, iNOS, and CSF1R. **j** Quantitative analysis of IL-1β, TNF-α, iNOS, and CSF1R (*n* = 3). **k** Representative western blots of SIRT3 in BV2 cells. **l** Quantitative analysis of SIRT3 in BV2 cells (*n* = 3). **m** Representative western blots of p-NF-kB, NF-kB, p-MAPK, and MAPK in BV2 cells. **n** Quantitative analysis of p-NF-kB and p-MAPK in BV2 cells (*n* = 3). **o** Representative western blots of SIRT3 in mouse brains. **p** Quantitative analysis of SIRT3 in mouse brains (*n* = 3). **q** Representative western blots of p-NF-kB, NF-kB, p-MAPK, and MAPK in mice. **r** Quantitative analysis of p-NF-kB and p-MAPK levels in mouse brains (*n* = 3). The data are presented as the means ± SEMs. Statistical significance is indicated as follows: compared with the control group: *p < 0.05; compared with the PA group: ^∆^*p* < 0.05; compared with the PA + AA 5 µM group: ^#^*p* < 0.05; and compared with the PA + AA 10 µM group: ^&^*p* < 0.05; *p < 0.05, **p < 0.01

Then, BV2 cells were pretreated with AA for 24 h and stimulated with PA (175 mM) for 8 h. We found that AA (10 and 20 μM) suppressed morphological changes in PA-induced BV2 cells (Supplementary Fig. 1d). Then, we examined the expression levels of microglial markers and CSF1R. The quantitative qPCR results showed that TNF-α, IL-1β, IL-6, iNOS, and CD16/32 expression levels in PA-treated cells were significantly higher than those in untreated cells and cells pretreated with AA (*p* < 0.05, Fig. 5d–h). Similarly, TNF-α, IL-1β, IL-6, iNOS, and CSF1R protein levels in PA-induced cells were significantly higher than those in cells that were pretreated with AA and untreated cells (*p* < 0.05, Fig. 5i, j). In addition, ELISA was used to examine IL-1β and TNF-α levels in the supernatant and revealed that the PA group had increased IL-1β and TNF-α levels compared with the control and PA + AA groups (*p* < 0.05, Fig. 5b, c). For immunofluorescence analysis, we labeled CD16/32 (an M1 macrophage surface marker) to examine the M1 phenotype in BV2 cells, and the results showed that PA-induced cells had increased membrane CD16/32 expression (Fig. 5a). These results indicate that PA induces M1 polarization in BV2 cells and that AA attenuates PA-induced BV2 cell activation. Notably, there was no significant difference in the therapeutic effects of 10- and 20-μM AA. Therefore, in subsequent experiments, AA 10-μM was selected as the final concentration.

### AA Enhanced SIRT3 Expression and Inhibited the NF-κB/MAPK Pathway in HFD-Fed Mice and PA-Induced BV2 Cells

Research has demonstrated that the NAD-dependent deacetylase sirtuin-3 (SIRT3) inhibits the NF-κB and MAPK signaling pathways to regulate the inflammatory process [19, 20]. To further elucidate the mechanisms of positive AA-mediated effects against brain aging, especially anti-inflammatory effects, we examined the expression levels of SIRT3, NF-κB, and MAPK in mouse brain tissue and BV2 cells. Compared with mice that were fed a normal diet, chronic HFD exposure significantly decreased the expression of SIRT3 in the brain and activated the NF-κB and MAPK pathways, as indicated by increased levels of p-NF-κB and p-MAPK (*p* < 0.01, Fig. 5k–m). In comparison to untreated HFD mice, AA-treated mice exhibited increased levels of SIRT3 (*p* < 0.05, Fig. 5o, p) and reduced levels of NF-κB- and MAPK-related proteins (*p* < 0.05, Fig. 5q, r). The results of the cell experiment were consistent with those of the animal experiment. The results suggested a clear benefit of AA treatment in enhancing SIRT3 activity and reducing NF-κB and MAPK signaling in the brains of HFD mice and in PA-induced BV2 cells. A previous study reported that SIRT3 participates in energy metabolism and anti-inflammatory and antiaging processes by regulating the NF-κB/MAPK signaling pathway. We further explored whether AA inhibited PA-induced BV2 activation by regulating the SIRT3-NF-κB/MAPK pathway.

### AA Inhibited PA-Induced BV2 Activation by Targeting the SIRT3-NF-κB/MAPK Pathway

**Fig. 6:**
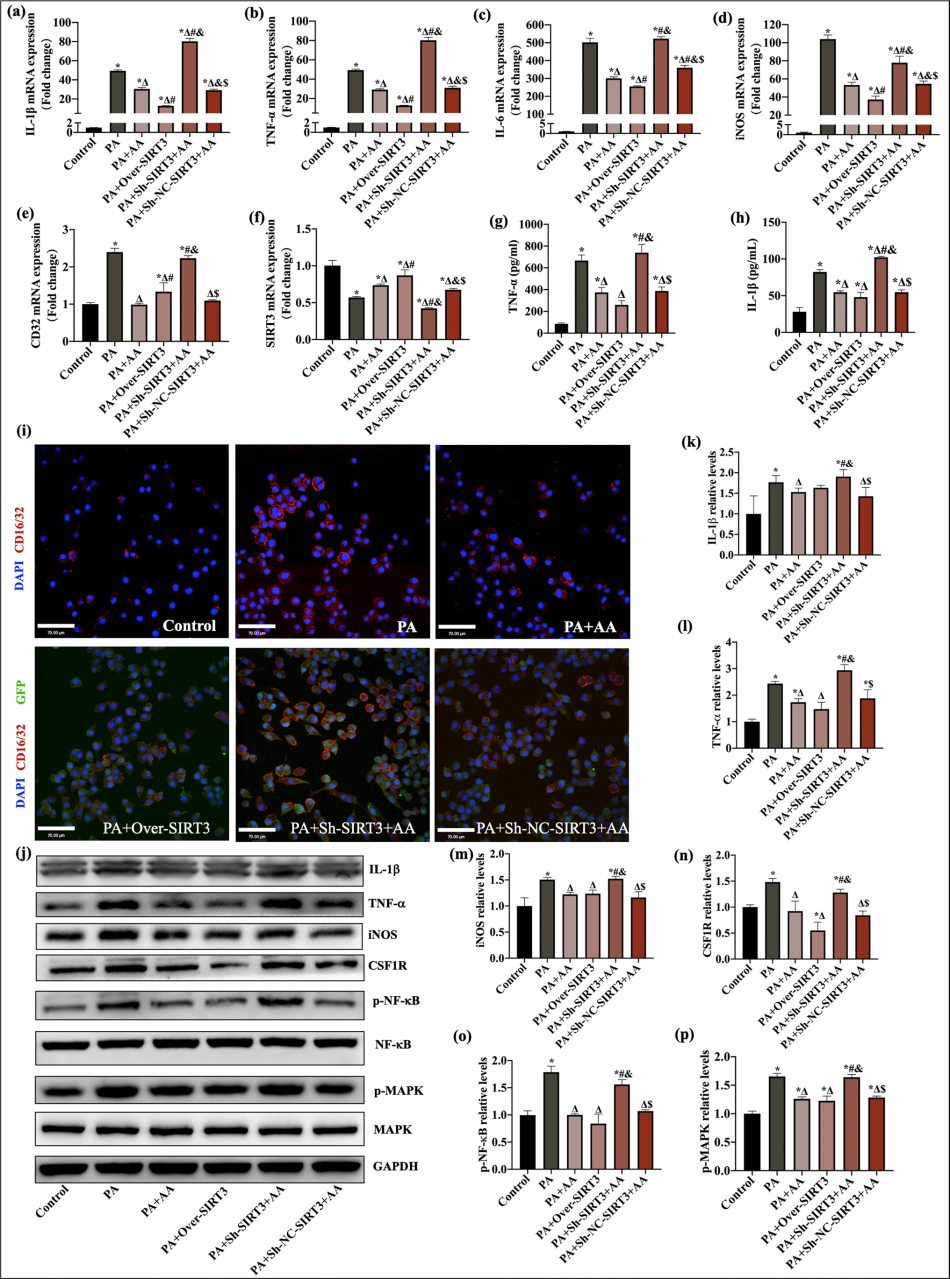
SIRT3 mediated the effects of AA on inhibiting BV2 activation and regulating the NF-κB and MAPK pathways. **a**–**f** The mRNA expression of IL-1β, TNF-α, IL-6, CD32, iNOS, and SIRT3 in each group (*n* = 3). **g**, **h** TNF-α and IL-1β levels in the culture supernatant were measured by ELISA (*n* = 3). **i** Representative immunostaining of the M1 macrophage surface marker CD16/32 (red) in each group. (**j**) Representative western blots of IL-1β, TNF-α, iNOS, CSF1R, p-NF-kB, NF-kB, p-MAPK, and MAPK. **k**–**p** Quantitative analysis of IL-1β, TNF-α, iNOS, CSF1R, p-NF-kB, NF-kB, p-MAPK, and MAPK (*n* = 3). The data are presented as the means ± SEMs. Statistical significance is indicated as follows: compared with the control group, *p < 0.05; compared with the PA group, ^∆^*p* < 0.05; compared with the PA + AA group, ^#^*p* < 0.05; compared with the PA + Over-SIRT3 group, ^&^*p* < 0.05; and compared with the PA + Sh-SIRT3 + AA group, ^$^*p* < 0.05. Over, overexpression; Sh, short hairpin RNA; NC, negative control (empty vector); GFP, green fluorescent protein

To analyze the role of SIRT3 in PA-induced M1 polarization in BV2 cells, we performed stable knockdown and overexpression of SIRT3 in BV2 cells using lentiviruses. Supplementary Fig. 2 shows that the second short hairpin RNA (Sh2-SIRT3) oligonucleotide successfully suppressed SIRT3 expression and that the overexpression lentivirus (Over-SIRT3) increased SIRT3 expression compared with that in the control group (*p* < 0.05, Supplementary Fig. 2). As shown in Fig. 6, in the Over-SIRT3 group, relative TNF-α, IL-1β, IL-6, iNOS, and CD16/32 mRNA expression were decreased in contrast to that in the PA group (*p* < 0.05, Fig. 6a–e). The protein levels of IL-6, iNOS, and CSF1R were decreased compared to those in the PA group (*p* < 0.05, Fig. 6j–n). ELISA analysis of IL-1β and TNF-α levels in the supernatant revealed that the Over-SIRT3 group had reduced IL-1β and TNF-α levels compared with the PA group (*p* < 0.05, Fig. 6g, h). Immunofluorescence analysis showed that SIRT3 overexpression decreased membrane CD16/32 expression when the cells were exposed to PA (Fig. 6i). In addition, SIRT3 overexpression suppressed the PA-induced NF-κB and MAPK pathways in contrast to that in the PA group (*p* < 0.05, Fig. 6j, o, p). These results demonstrated that SIRT3 overexpression markedly inhibited PA-induced M1 activation in BV2 cells, which was similar to the effect of AA treatment. The therapeutic effects of AA on inhibiting PA-induced M1 activation in BV2 cells were significantly reversed by sh-SIRT3. These results suggested that SIRT3 inhibited the NF-κB/MAPK pathway (*p* < 0.05, Fig. 6), which mediated AA’s effects on PA-induced BV2 cell activation.

### BV2-Conditioned Medium Containing AA Protected the Integrity of the Endothelial Cell Barrier by Reducing TJ Degradation

**Fig. 7:**
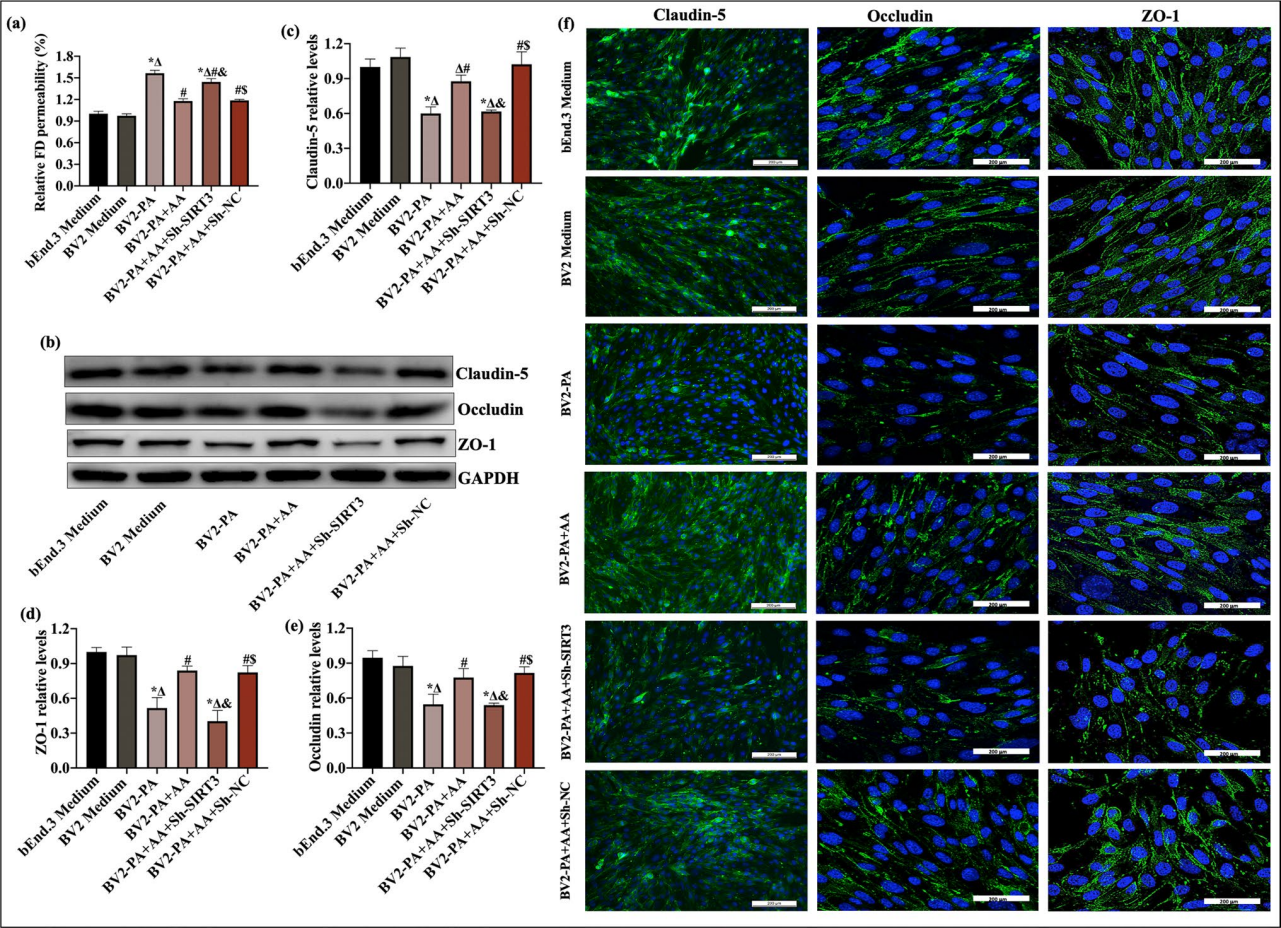
Conditioned medium from BV2 cells treated with AA attenuated bEnd.3 cell barrier breakdown. After being cultured under normal culture conditions to 70–80% confluence, bEnd.3 cells were cultured with different BV2-conditioned media for another 24 h. The groupings in experiments involving BV2-conditioned medium were based on BV2 treatments. **a** Endothelial monolayer permeability was examined by FITC-dextran crossing bEnd.3 cells (*n* = 4). **b** Representative western blots of claudin-5, occludin, and ZO-1 in bEnd.3 cells. (**c**–**e**) Quantitative analysis of claudin-5, occludin, and ZO-1 in bEnd.3 cells (*n* = 3). **f** Representative immunostaining of tight junction proteins, including claudin-5, occludin, and ZO-1, in bEnd.3 cells. The data are presented as the means ± SEMs. Statistical significance is indicated as follows: compared with the bEnd.3 medium group, *p < 0.05; compared with the BV2 medium group, ^∆^*p* < 0.05; compared with the BV2-PA group, ^#^*p* < 0.05; compared with the BV2-PA + AA, ^&^*p* < 0.05; and compared with the BV2-PA + AA + Sh-SIRT3 group, ^$^*p* < 0.05

Previous studies have shown that BBB dysfunction is an early driver of brain dysfunction. Neuroinflammation induced by a HFD directly or indirectly disrupts the integrity of the BBB. This study examined whether PA-induced BV2 activation increased endothelial cell permeability and further evaluated the effect of AA on protecting the integrity of the BBB. After BV2 cells were exposed to PA or pretreated with AA and then exposed to PA, the medium of bEnd.3 cells was replaced with BV2-conditioned medium (BCM). As shown in Fig. 7a, BCM from the normal BV2 group did not affect bEnd.3 cell permeability (*p* > 0.05). BCM from the PA group significantly increased the permeability of confluent bEnd.3 cells (*p* < 0.05). When bEnd.3 cells were cultured with BCM from the PA + AA group, the permeability of bEnd.3 cells significantly decreased in contrast to the PA group (*p* < 0.05), while the knockdown of SIRT3 (Sh-SIRT3) significantly neutralized the protective effect of AA (*p* < 0.05).

We also examined the levels of TJ proteins in bEnd.3 cells cultured with different BCMs. The levels of important TJs in bEnd.3 (ZO-1, occludin, and claudin-5) were significantly reduced in the BV2-PA group compared with the BV2 medium group (*p* < 0.05, Fig. 7b–f), but the levels were restored in the BV2-PA + AA group (*p* < 0.05). It should be noted that the effect of AA on BV2 cells was abolished by SIRT3 knockdown (*p* < 0.05).

### BV2-Conditioned Medium Containing AA Ameliorated HT22 Cell Apoptosis

**Fig. 8:**
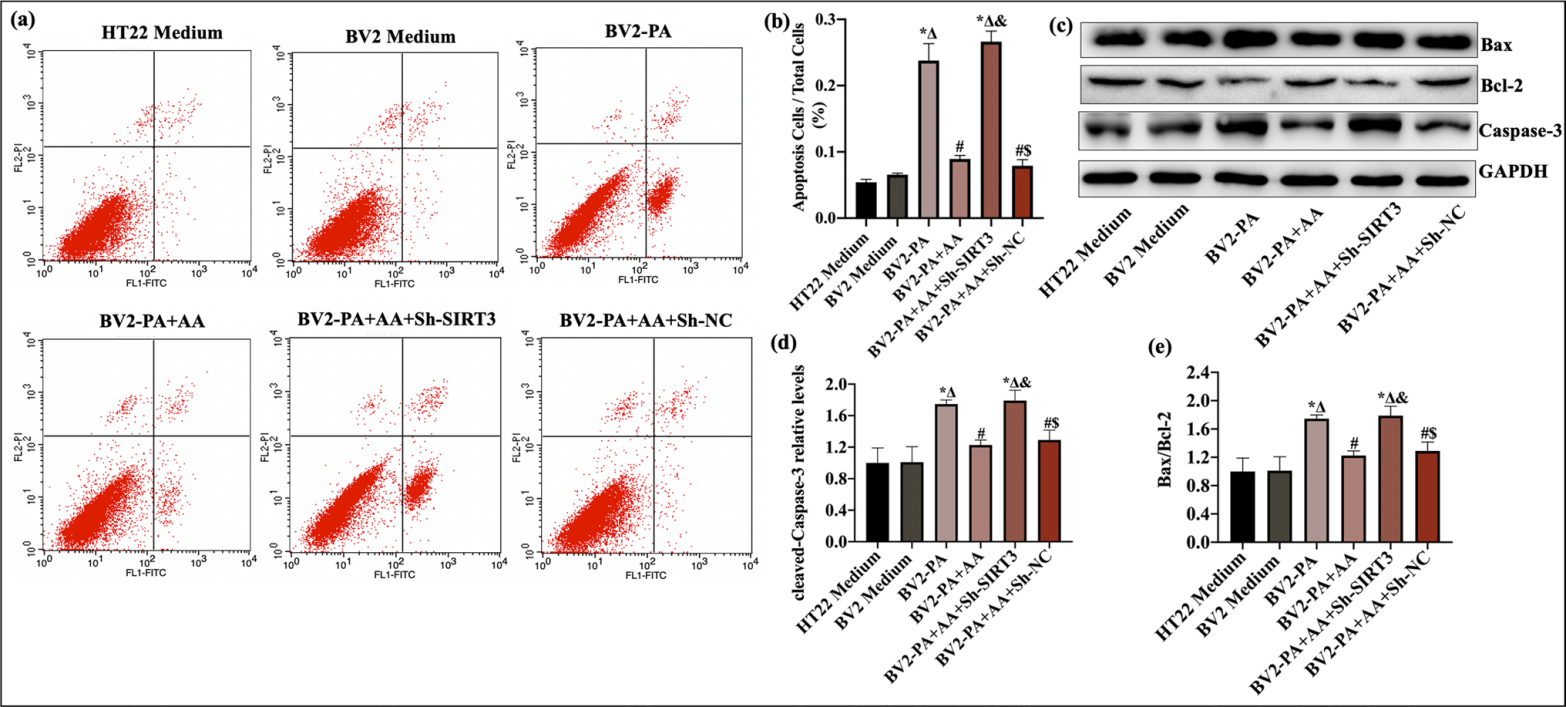
Conditioned medium from BV2 cells treated with AA reduced HT22 cell apoptosis. After being cultured under normal culture conditions to 70–80% confluence, HT22 cells were cultured with different BV2-conditioned media for another 24 h. The groupings in experiments involving BV2-conditioned medium were based on BV2 treatments. **a** Flow cytometric analysis of HT22 cells stained with annexin V-FITC/PI. **b** Quantitative analysis of the apoptosis rate in **a** (*n* = 3). **c** Representative western blots of cleaved caspase-3, Bcl-2, and Bax in HT22 cells. **d**, **e** Quantitative analysis of Bax/Bcl-2 and cle-caspase-3 expression in HT22 cells (*n* = 3). The data are presented as the means ± SEMs. Statistical significance is indicated as follows: compared with the HT22 medium group, *p < 0.05; compared with the BV2 medium group, ^∆^*p* < 0.05; compared with the BV2-PA group, ^#^*p* < 0.05; compared with the BV2-PA + AA group, ^&^*p* < 0.05; and compared with the BV2-PA + AA + Sh-SIRT3 group, ^$^*p* < 0.05

Inflammation-induced apoptosis plays a crucial role in neuronal injury and death in brains subjected to HFD-induced metabolic disorders. In this study, HT22 cells were used to observe the effect of inflammatory substances secreted by PA-induced BV2 cells on neuronal apoptosis. HT22 cell apoptosis was analyzed by flow cytometry and WB analysis. As shown in Fig. 8a, compared with that in the HT22 medium group, BCM from the BV2 medium group had no significant effect on the apoptosis rate of HT22 cells (*p* > 0.05). BCM from the PA group significantly increased the expression of caspase-3 and decreased the Bcl2/Bax ratio in HT22 cells compared to normal BV2 medium (*p* < 0.05). BCM containing AA reversed the effects of by PA (*p* < 0.05). In addition, SIRT3 knockdown abolished the protective effect of AA against apoptosis in HT22 cells (*p* < 0.05). Overall, these results indicated that BV2-conditioned medium containing AA ameliorated HT22 cell apoptosis induced by BCM with PA.

## Discussion

As with other organ systems, the functional capabilities of the brain decline progressively with age, which manifests as decrements in learning, memory, attention, decision-making speed, sensory perception, and motor coordination [20, 21]. There are dramatic and significant variabilities in both the rate and severity of these alterations. Emerging evidence has revealed that sedentary and overindulgent lifestyles and metabolic perturbations accelerate brain aging. HFDs have been firmly shown to promote different aspects of brain aging [2, 22, 23] Several cross-sectional and longitudinal clinical studies have shown that long-term high-fat diets in young [24], middle-aged [25], and elderly people [26, 27] can impair cognitive function [28]. Similarly, in rodents, HFD-induced obese animals have reduced spatial learning ability and hippocampal plasticity [29] and increased social disorders [30]. With the large number of overweight and obese individuals caused by a HFD and the aging of the population, the impact of HFDs on aging-related brain structure and function has attracted increasing attention.

AA has been reported to effectively reduce high fat-induced obesity, inhibit liver steatosis, improve lipid and glucose metabolism, and reduce the inflammatory state of adipose tissue in mice, making AA a promising treatment for obesity and obesity-related metabolic disorders [12, 13]. However, whether it exerts neuroprotective effects against HFD-induced pathological brain aging has not yet been reported. This study demonstrated that 12 weeks of a HFD impaired cognitive function in middle-aged mice. The cognitive functional changes include decreased learning and memory ability associated with spatial position and orientation in the Morris water maze and decreased recognition memory in the novel object recognition test. HFD mice treated with AA showed better cognitive function than age-matched HFD mice that received no treatment.

Neuroinflammation is a common pathological center in acute and chronic neurological diseases. Although the underlying mechanism by which a HFD affects pathological brain aging is still unclear, previous studies have shown that neuroinflammation plays an important role in diet-induced brain dysfunction [31, 32]. HFD induces a neuroinflammatory response mediated by activation of microglia in brain tissue, disrupting neurovascular structure and function and leading to pathological brain aging. Microglia have been widely shown to participate in the inflammatory response in the brain induced by HFD. Reactive microglia can acquire different phenotypes based on the activation stimuli encountered. Studies have shown that under HFD conditions, exposure to proinflammatory cytokines, such as IL-1β, IL-6, and TNF-α, or fatty acids polarized M1 microglia that could initiate a proinflammatory response [33]. The hippocampus and cortex of experimental animals showed neuroinflammation and gliosis and increased expression of markers associated with nerve damage in rodents [1, 34, 35]. In line with these results, in our study, 12 weeks of a HFD resulted in microglial activation in the cortex and hippocampus of mice. The M1 microglial activation markers TNF-α, IL-1β, and iNOS were increased in the brains of HFD-fed mice. WB analysis showed that colony-stimulating factor 1 receptor (CSF1R), which is necessary for microglial viability, was also increased. These changes induced by HFD were inhibited by AA. MRS analysis showed that mice exposed to a long-term HFD exhibited neurochemical modifications in the hippocampus and cortex and exhibited hippocampal-dependent memory impairment [36, 37]. Prior studies have also suggested that MRS could be promising for noninvasive evaluation of neuroinflammation [15, 17, 38]. The activation of astrocytes and microglia in brain diseases, leading to neuroinflammation, is often associated with an increase in mI and Cho [15, 38]. These substances have higher concentrations in glial cells than in neurons. Another study suggests that lipid and macromolecule levels could potentially serve as biomarkers for microglial activation, as they found that the activation of microglia induced by LPS in wild type mice is associated with a persistent increase in the levels of ML0.9 [17]. As shown in Fig. 2, we evaluated the changes in mI, Cho, and ML0.9, which have been reported in response to neuroinflammation or microglia activation. The results indicated that HFD exposure changed brain metabolic profiles associated with inflammatory processes and that AA reversed HFD-induced abnormal metabolism.

HFD triggers microglial M1 activation to damage not only the cortex, hippocampus, and other brain tissue but also the BBB. These interconnected cascades continue to damage the neurovascular unit, leading to pathological brain aging [36, 39]. Previous studies have shown that neuroinflammation plays an important role in diet-induced brain dysfunction [31, 32]. Some adipocytokines and inflammatory mediators in the peripheral and circulatory system caused by a HFD can enter the brain through the BBB, which subsequently activates inflammatory responses in the brain and further enhances neuronal excitability and increases BBB permeability to various molecules [40, 41]. Therefore, we examined HFD-induced damage to the neurovascular structure. Tight junctions (TJ), present between the cerebral endothelial cells, form a diffusion barrier, which selectively excludes most blood-borne substances from entering the brain. The expression and arrangement of TJ proteins play a crucial role in the normal functioning of the BBB. In this study, compared with normal diet-fed mice, HFD-fed mice exhibited fewer and irregular hippocampal neurons, decreased dendritic spines of cortical and hippocampal neurons, increased expression of apoptotic proteins, and decreased expression of TJ proteins ZO-1, occludin and claudin-5. HFD-fed mice treated with AA showed increased hippocampal neurons and dendritic spines in hippocampal and cortical region neurons. The data were also confirmed by the MRS scan of the cortex and hippocampus. Reduced NAA concentrations in the hippocampus of HFD mice are a marker of damaged neuronal integrity [14]. AA treatment resulted in increased NAA concentrations in the hippocampus. In addition, AA inhibited the degradation of TJ proteins ZO-1, occludin and claudin-5 in the brains of HFD mice. Our findings in this study showed that AA inhibited neurovascular damage caused by HFD, which is one of the manifestations by which AA improves cognitive function.

Because microglia-associated chronic neuroinflammation plays an important role in the pathophysiology of HFD-induced accelerated brain aging, we next examined the effect of AA on inhibiting palmitic acid (PA)-induced BV2 cell activation. Among FAs, PA is an abundant saturated FA that is present in the human body and closely linked to metabolic diseases. We found that 175-µM PA markedly activated BV2 cells. We verified that 10-µM AA significantly attenuated inflammatory responses in PA-induced BV-2 cells. This is the first report of the inhibitory effect of AA on microglial activation.

The NAD-dependent deacetylase sirtuin-3 (SIRT3), which is a member of the mammalian sirtuin family, is the main deacetylase in mitochondria and is expressed in metabolically active tissues such as the brain, fat, and muscle. Relevant studies have shown that the downregulation of SIRT3 is a key pathological factor of metabolic syndrome [37, 42]. In addition, SIRT3 is involved in inflammation regulation. Energy metabolism disorders and neuroinflammation caused by SIRT3 deficiency in metabolic diseases can cause cognitive dysfunction [43]. It was suggested that SIRT3 suppressed the inflammatory response through the NF-κB and MAPK signaling pathways [20, 43]. NF-κB is involved in cellular responses to stimuli such as stress, cytokines, and free radicals and is implicated in the processes associated with synaptic plasticity and memory. A reduction in NF-κB deacetylation leads to the upregulation of target genes of proinflammatory cytokines, enzymes, and cell adhesion molecules, thereby promoting inflammation; and SIRT3 can deacetylate NF-κB and attenuate inflammation [44]. MAPK signaling has also been shown to be a driving factor for the inflammation mediated by BV2 microglia stimulated by LPS [19]. SIRT3 downregulates the MAPK pathway and mediates TNF-α inhibition in U937 cells [45]. Therefore, regulating the SIRT3-NF-κB/MAPK pathway may be a strategy to inhibit the activation of microglia.

To further explore the mechanism by which AA inhibits the activation of microglia, we examined the levels of SIRT3, phosphorylated NF-κB p65 (p-NF-κB), and phosphorylated MAPK (p-MAPK) in the brains of mice and BV2 cells. We found that a HFD significantly reduced the expression of SIRT3 and increased p-NF-κB and p-MAPK expression in mouse brains, and the same change was observed in PA-induced BV2 cells, while AA treatment abrogated this effect. Overexpression of SIRT3 and AA inhibited microglial activation and suppressed p-NF-κB and p-MAPK expression. Conversely, SIRT3 knockdown abolished the therapeutic effect of AA on PA-induced BV2 activation and increased the levels of p-NF-κB and p-MAPK. This finding suggests that SIRT3 can inhibit the NF-κB/MAPK pathway, which mediates the effects of AA on microglial activation.

As previously mentioned, HFD-induced chronic inflammation aggravates neurons and BBB damage, contributing to cognitive impairment [36, 39]. The BBB is a dynamic specialized structure composed of brain endothelial cells that are tightly connected with each other through TJs. In our study, AA treatment suppressed HFD-induced TJ (ZO-1, occludin, and claudin-5) degradation. In parallel, the in vitro experiment revealed that the inhibitory effect of AA on PA-induced BV2 cell activation protected the integrity of the bEnd.3 cell barrier. The hippocampus is one of the most susceptible structures to a HFD. We found that long-term HFD consumption reduced the number of hippocampal neurons and the complexity of the dendrites, while AA treatment ameliorated these pathological changes. In vitro, our results showed that BV2-conditioned medium (BCM) and AA treatment ameliorated HT22 cell apoptosis induced by BCM plus PA. Based on these findings, we considered that AA could inhibit HFD-induced TJ degradation and neuronal injury by inhibiting BV2 cell activation.

In conclusion, our results demonstrated that the therapeutic effect of AA on HFD-induced unhealthy brain aging in mice is linked to the inhibition of neuroinflammation. In addition, AA can inhibit PA-induced microglial activation by upregulating SIRT3 and downregulating the NF-κB/MAPK pathway. Our results revealed that inhibiting BV2 microglial activation via the SIRT3-NF-κB/MAPK signaling pathway might be the mechanism by which AA facilitates functional benefits in HFD mice.

### Supplementary Information

Below is the link to the electronic supplementary material.

Supplementary file1 (DOCX 11398 KB)

## Data Availability

The datasets generated during and/or analyzed during the current study are available from the corresponding author on reasonable request.

## Abbreviations

AA: Alisol A
BBB: Blood-brain barrier
BCM: BV2-conditioned medium
bEnd.3: Mouse brain capillary endothelial cell
BMEC: Brain microvascular endothelial cells
BV2: BV-2 microglial cells
CCL2: Chemokine ligands 2
Cho: Choline
CNS: Central nervous system
Cr: Creatine
CSF1R: Colony-stimulating factor 1 receptor
DMEM: Dulbecco’s modified Eagle’s medium
DMSO: Dimethyl sulfoxide
ELISA: Enzyme-linked immunosorbent assay
FBS: Fetal bovine serum
GAPDH: Glyceraldehyde-3-phosphate dehydrogenase
h: Hour
HFD: High-fat diet
HT22: Mouse hippocampal cell line
Iba1: Ion calcium junction protein
IL-1β: Interleukin-1β
IL-6: Interleukin-1β
iNOS: Inducible NO synthase
LPS: Lipopolysaccharide
MAPK: Mitogen-activated protein kinase
mI: Myo-inositol
min: Minute
ML0.9: Macromolecules and lipids at 0.9 ppm
MOI: Multiplicity of infection
MWM: Morris water maze
NAA: N-acetylaspartate
NF-κB: Nuclear factor kappa light chain enhancer of activated B cells
NORT: Novel object recognition test
PA: Palmitate
PBS: Phosphate-buffered saline
PCR: Polymerase chain reaction
PVDF: Polyvinylidene difluoride
ROS: Reactive oxygen species
rpm: R/min
s: Second
SDS: Sodium dodecyl sulfate
ShRNA: Short hairpin RNA
SIRT3: Sirtuin 3
TJ: Tight junction
TNF-α: Tumor necrosis factor-α
WB: Western blot
ZO-1: Zonula occludens-1
