# Supplementary material for: Alisol A Exerts Neuroprotective Effects Against HFD-Induced Pathological Brain Aging via the SIRT3-NF-κB/MAPK Pathway
Source: Mol Neurobiol. 2023 Sep 2;61(2):753–71. doi: 10.1007/s12035-023-03592-5 (PMC10861652; doi:10.1007/s12035-023-03592-5)
Supplement: Supplementary file 1 — Supplementary file1 (DOCX 11398 KB) [file 12035_2023_3592_MOESM1_ESM.docx]

**Table S1**. Primers used for qPCR study

| Gene | Sequence (5'-3’) |
| --- | --- |
| TNF‐α-F (mus) | GCCGATGGGTTGTACCTTGT |
| TNF‐α-R (mus) | TCTTGACGGCAGAGAGGAGG |
| IL‐1β-F (mus) | GAAATGCCACCTTTTGACAGTG |
| IL‐1β-R (mus) | TGGATGCTCTCATCAGGACAG |
| IL-6-F (mus) | CTGCAAGAGACTTCCATCCAG |
| IL-6-R (mus) | AGTGGTATAGACAGGTCTGTTGG |
| CD32-F (mus) | TGTCACCATCACTGTCCAAGG |
| CD32-R (mus) | GATAATAACAATGGCTGCGAC |
| iNOS-F (mus) | GGAGTGACGGCAAACATGACT |
| iNOS-R (mus) | TCGATGCACAACTGGGTGAAC |
| GAPDH-F (mus) | ACGGCAAGTTCAACGGCACAG |
| GAPDH-R (mus) | GAAGACGCCAGTAGACTCCACGAC |
| sirt3-F1 (mus) | AGCTACATGCACGGTCTGTC |
| sirt3-R1 (mus) | AATGTCGGGTTTCACAACGC |
| sirt3-F2 (mus) | CAGAACATCGACGGGCTTGA |
| sirt3-R2 (mus) | TTCACAACGCCAGTACAGACA |
| sirt3-F3 (mus) | CTGTCTGTACTGGCGTTGTG |
| sirt3-R3 (mus) | AGGTCCCAAGAATGAGTAGCA |

**
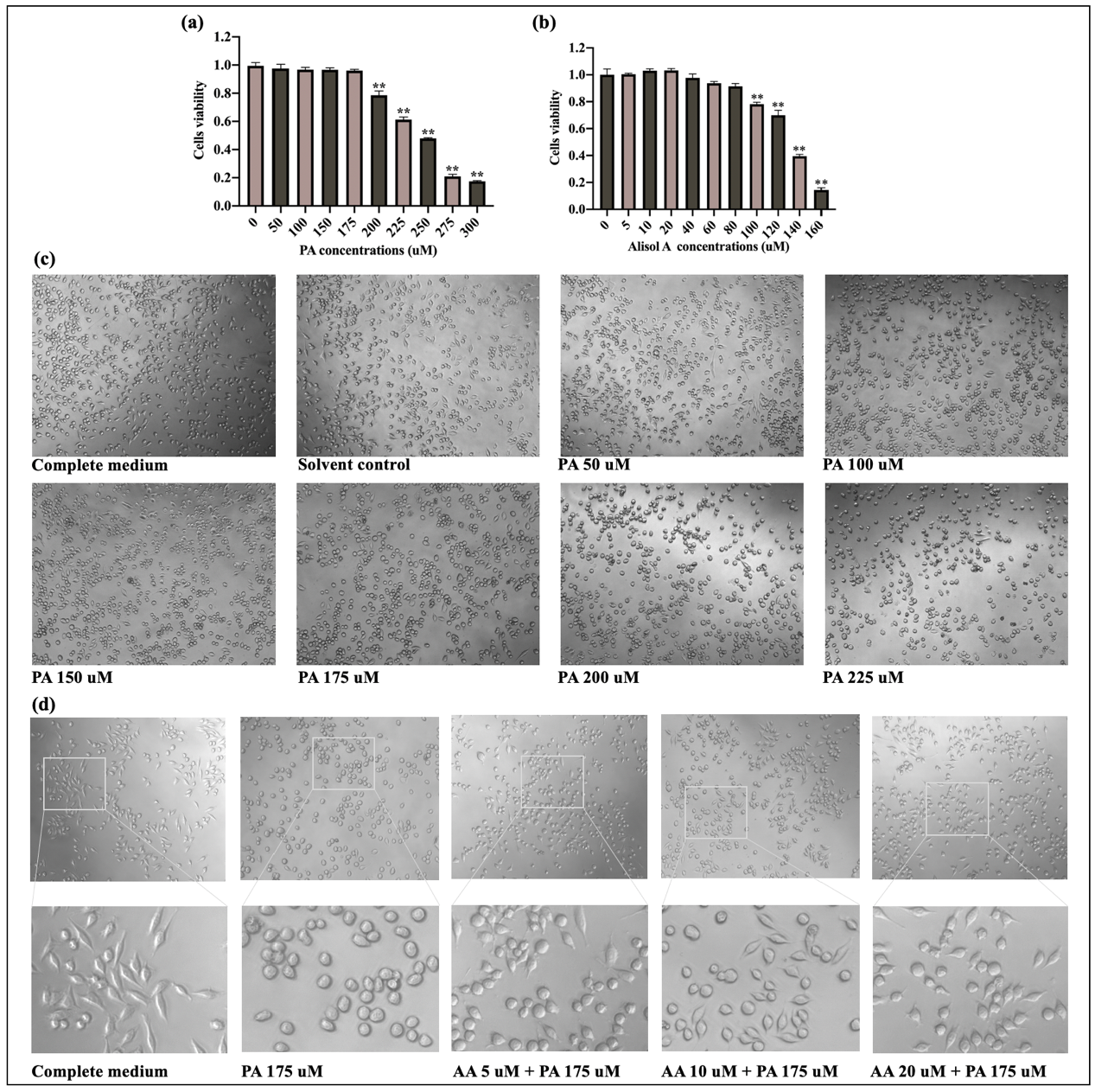
Fig. S1 AA reversed morphological variation of the PA-stimulated BV2 cells**

(a) BV2 cells in logarithmic growth phase were cultured in serum-free medium for 12 hours. Thereafter, cells were treated with PA (0-300 μM) for 8 hours and the cells viability were measured by using CCK-8 (n=6). (b) BV2 cells in logarithmic growth phase were treated with AA (0-160 μM) for 24 hours and the cells viability were measured by using CCK-8 (n=6). (c) The cells morphology was recorded by optical microscopy. (d) BV2 cells were pre-treated with AA (5, 10, 20 μM) for 24 hours and stimulated with PA for 8 hours. Then, the cells morphology was recorded by optical microscopy. Data presented as means ± SEMs. Statistical significance indicated as: **p*< 0.05, ***p*< 0.01 compared with the concentration (0 μM). Abbreviation: AA=Alisol A; PA= palmitate.

**
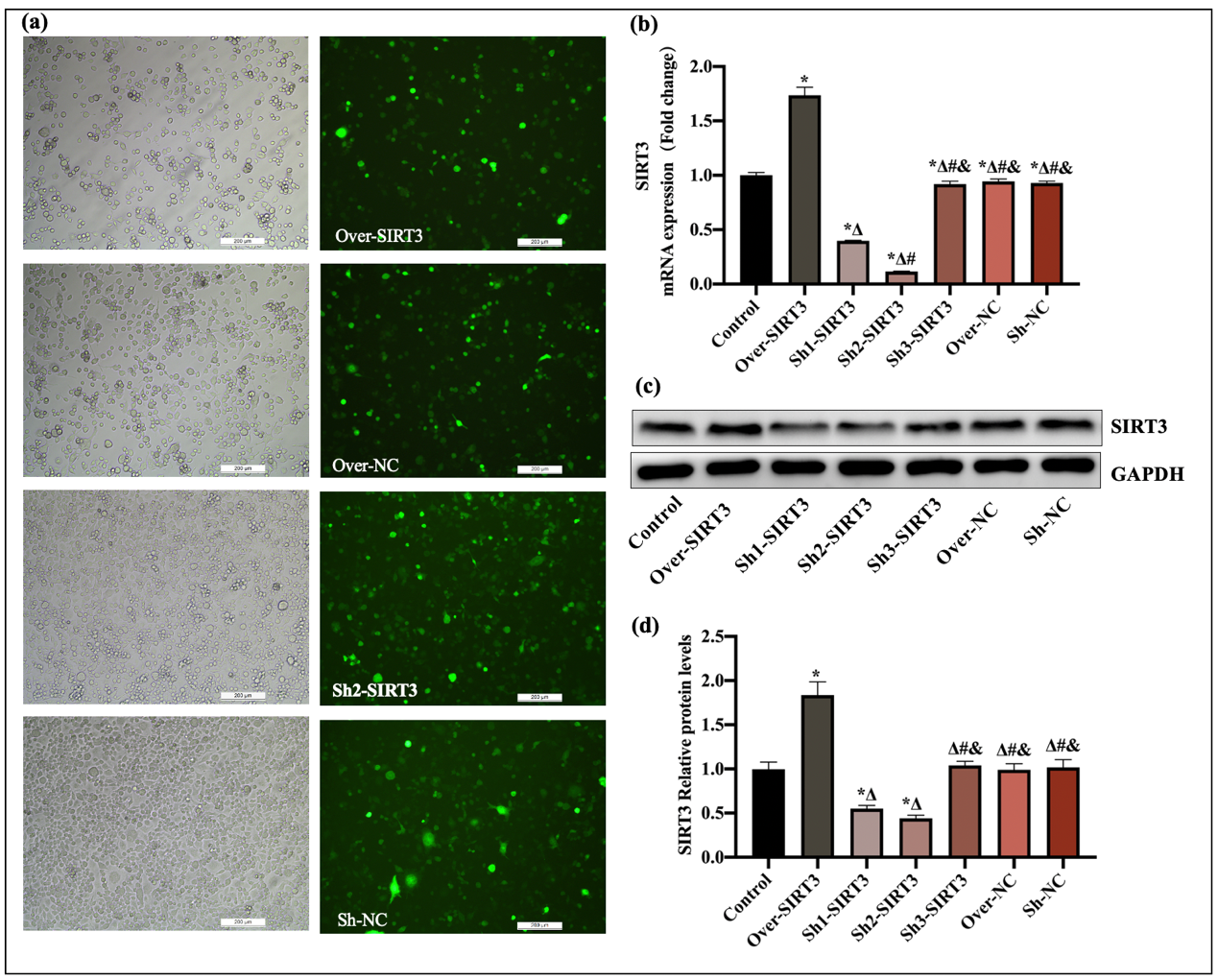
Fig. S2 Lentivirus-mediated stable knock-down and over-expression of SIRT3**

To generate stable BV2 cell lines that knock-down or over-expression of SIRT3, three non-overlapping anti-SIRT3 short hairpin RNA (ShRNA) oligonucleotides and lentivirus-mediated over-expression of SIRT3 were transfected into BV2 cells. Fig.S2 illustrated that the second short hairpin RNA (Sh2-SIRT3) oligonucleotides successfully suppressed SIRT3 expression and overexpression lentivirus (Over-SIRT3) increased SIRT3 expression effectively. (a)Transfection efficiency of SIRT3 detected by fluorescence microscope. (b) mRNA expression of SIRT3 expression in each group (n=3). (c) Representative western blots of SIRT3. (d) Quantitative analysis of SIRT3 (n=3). Data presented as means ± SEMs. Statistical significance indicated as: Compared with the control group **p*<0.05; Compared with the Over-SIRT3 group ^∆^*p*<0.05; Compared with the Sh1-SIRT3 group ^#^*p*<0.05; Compared with the Sh2-SIRT3 group ^&^*p*<0.05. Abbreviation: Over=over expression; Sh=short hairpin RNA; NC=Negative control (that is empty vector).
